# Bioinformatic analysis and experimental validation of the potential gene in the airway inflammation of steroid-resistant asthma

**DOI:** 10.1038/s41598-023-35214-4

**Published:** 2023-05-19

**Authors:** Chaochao Wei, Yang Wang, Chengping Hu

**Affiliations:** 1 Department of Pulmonary and Critical Care Medicine, Hainan General Hospital, Haikou, People’s Republic of China; 2 Department of Pulmonary and Critical Care Medicine, Affiliated Hainan Hospital of Hainan Medical University, Haikou, People’s Republic of China; 3 Department of Oncology, Xiangya Hospital Central South University, Changsha, People’s Republic of China; 4 Key Laboratory of Emergency and Trauma of Ministry of Education, Hainan Medical University, Haikou, 571199 People’s Republic of China; 5 Department of Respiratory Medicine (Department of Respiratory and Critical Care Medicine), Xiangya Hospital, Central South University, Changsha, 410008 Hunan People’s Republic of China; 6 National Clinical Research Center for Geriatric Disorders, Xiangya Hospital, Central South University, Changsha, 410008 Hunan People’s Republic of China

**Keywords:** Asthma, Genetics, Immunology, Respiratory tract diseases

## Abstract

Steroid-resistant asthma is a troublesome clinical problem in public health. The pathogenesis of steroid-resistant asthma is complex and remains to be explored. In our work, the online Gene Expression Omnibus microarray dataset GSE7368 was used to explore differentially expressed genes (DEGs) between steroid-resistant asthma patients and steroid-sensitive asthma patients. Tissue-specific gene expression of DEGs was analyzed using BioGPS. The enrichment analyses were performed using GO, KEGG, and GSEA analysis. The protein–protein interaction network and key gene cluster were constructed using STRING, Cytoscape, MCODE, and Cytohubba. A steroid-resistant neutrophilic asthma mouse model was established using lipopolysaccharide (LPS) and ovalbumin (OVA). An LPS-stimulated J744A.1 macrophage model was prepared to validate the underlying mechanism of the interesting DEG gene using the quantitative reverse transcription-polymerase chain reaction (qRT-PCR). A total of 66 DEGs were identified, most of which were present in the hematologic/immune system. Enrichment analysis displayed that the enriched pathways were the IL-17 signaling pathway, MAPK signal pathway, Toll-like receptor signaling pathway, and so on. DUSP2, as one of the top upregulated DEGs, has not been clearly demonstrated in steroid-resistant asthma. In our study, we observed that the salubrinal administration (DUSP2 inhibitor) reversed neutrophilic airway inflammation and cytokine responses (IL-17A, TNF-α) in a steroid-resistant asthma mouse model. We also found that salubrinal treatment reduced inflammatory cytokines (CXCL10 and IL-1β) in LPS-stimulated J744A.1 macrophages. DUSP2 may be a candidate target for the therapy of steroid-resistant asthma.

## Introduction

Asthma is commonly recognized as a chronic inflammatory respiratory disease, accompanied by airway reversible obstruction and respiratory symptoms, including cough, shortness of breath, and chest tightness^1^. Steroid inhalation is widely used for alleviating airway inflammation in asthma and reducing asthma exacerbations^2^. Despite high doses of steroid inhalation or systemic steroid usage, about 10% of asthma patients develop steroid resistance and lose control of symptoms^3,4^, whose phenotype is usually neutrophilic dependent^5^. The steroid is the main therapy for asthma but has an unsatisfactory response in neutrophilic asthma patients^6^. It was reported that IL-17A induced Th17 cells to recruit neutrophils in the airway of steroid-resistant asthma and Th17 cells played an important part in steroid-resistant asthma with neutrophilic airway inflammation^7^. A previous study suggested that steroid inhibited Th1/Th2‐related inflammatory cytokine production^8^, but failed to affect the production of Th17-related cytokines in steroid-resistant asthma^9,10^. The mechanisms of steroid resistance related to the IL-17A pathway remained to be elusive. The multiple mechanisms underlying the etiology of steroid-resistant asthma are complex^11,12^.

Given that steroid-resistant asthma is a complex clinical problem in public health, it is urgent to find an effective therapeutic strategy for steroid-resistant asthma. Therefore, well-established computational data-mining strategies were developed to explore the responsible genes for steroid-resistant asthma. Microarray dataset GSE7368 of bronchoalveolar lavage fluid (BALF) cells was used to identify the differentially expressed genes (DEGs) between steroid-resistant (SR) asthma patients and steroid-sensitive (SS) asthma patients, which aimed to provide a better understanding of the genetic etiology of steroid-resistant asthma.

## Materials and methods

All the experimental protocols were approved by the Ethics Committee of Xiangya Hospital, Central South University (committee Reference Number: 201803691). Methods used in experimental animals in the study were approved by the Central South University Experimental Animal Ethics Committee of Xiangya Hospital and complied with ARRIVE guidelines and American Veterinary Medical Association (AVMA) Guidelines for the Euthanasia of Animals (2020). The datasets generated from human participants can be found in the GEO dataset: https://www.ncbi.nlm.nih.gov/geo/query/acc.cgi?acc=GSE7368, which was performed following the ethical standards laid down in the 1964 Declaration of Helsinki and its later amendments.

### Microarray data

The microarray dataset GSE7368, constructed by Goleva et al., was retrieved from the Gene Expression Omnibus (GEO, https://www.ncbi.nlm.nih.gov/geo/), which allows researchers to search and download expression data for analysis based on the GPL570 [HG-U133_Plus_2] Affymetrix Human Genome U133 Plus 2.0 Array platform. The experiment contained gene array studies of BALF cells, consisting of 3 steroid-resistant (SR) asthma patients and 3 steroid-sensitive (SS) asthma patients. The data were normalized using the gcrma package (version 2.0.0)^13^. All analyses were performed in the R environment (version 3.6.2). The probes that have no expression in most of the samples were removed. The probes were annotated as gene symbols based on the annotation information of the GPL570 platform. An expression value for every gene was acquired based on mean expression estimates. As a result, we obtained the gene expression matrix.

### Differential expression analysis

Differential expression analysis was performed to identify the DEGs using a limma package (version 3.42.2)^14^. *P* values were calculated using the t-tests. The DEGs were determined according to the following criteria: (1) a |log2 (fold-change)|> 1 and (2) a *P* value < 0.05. The volcano plot and the heatmap for the DEGs were constructed using ggplot2 (version 3.3.1) and pheatmap (version 1.0.12) packages^15,16^. We selected the top 6 upregulated DEGs to create a violin plot.

### Tissue-specific expressed gene analysis

Online database BioGPS (http://biogps.org) (version 94eefe6) was used to analyze the tissue-specific expressed DEG genes. The genes identified as highly tissue-specific genes were similar to the previous study described, with more than > 30 multiples of the median (MoM) and less than 1/3 of the highest expressions in the second highest level^17^.

### Functional enrichment analysis of DEGs

The functional enrichment analysis of DEGs was performed using the clusterProfiler package (version 3.14.3)^18^. The enrichment analyses were performed for predicting protein functions. The biological process (BP), cellular component (CC), and molecular function (MF) of DEGs were identified using the enrichGO function in the clusterProfiler package. The pathway enrichment was identified using the enrichKEGG function in the clusterProfiler package.

### Gene set enrichment analysis

We performed GSEA analysis using GSEA software (version 4.0.3)^19^. KEGG enrichment pathways were identified with the following criteria: a *P* value < 5% and a false discovery rate (FDR) < 25% for every analysis.

### Protein–protein interaction (PPI) network analysis

Search Tool for the Retrieval of Interacting Genes (STRING; http://string-db.org) (version 10.0)^20^ was used to predict and construct the protein–protein interactions (PPI) network of DEGs. DEGs in the PPI network arrived at a minimum required interaction score > 0.4. The nodes indicate genes, and the edges indicate the associations between genes. The PPI network was visualized using Cytoscape (version 3.7.1). Besides, Molecular Complex Detection (MCODE) (version 1.6) in Cytoscape was used to assess the key clustered module with a degree cutoff of 2, a node score cutoff of 0.2, and a K-Core of 2^21^. The clustered module of the top 10 genes was identified using CytoHubba (version 0.1) in Cytoscape with the MCC method^22^.

### Animal experiments

Male C57BL/6 mice (6–8 weeks of age) were used to develop a steroid-resistant neutrophilic asthma (NA) model as described by previous studies with minor modification^23–26^. All mice were divided into five groups (n = 4 per group) randomly: Control group, NA group, NA + salubrinal group, NA + Dex group, and Control + salubrinal group. Briefly, ovalbumin (OVA, 100 μg) and lipopolysaccharides (LPS, 10 μg) in 50 μl PBS were intratracheally delivered to the mice under anesthesia with subcutaneous pentobarbital on day 0 and day 6. Then the mice were challenged with 5% aerosolized OVA for 40 min on day 13. The mice were sacrificed on day 14, and the lungs were extracted for histology analysis using hematoxylin and eosin (H&E) and Masson staining^27,28^. In some groups, the LPS/OVA-sensitized mice were injected intraperitoneally with dexamethasone (Dex, 1 mg/kg) or salubrinal (2 mg/kg) before the OVA challenge on day 13. The control mice were administered intratracheally with 50 μL PBS, then challenged with PBS for 40 min on day 13. In another group, the control mice were injected intraperitoneally with salubrinal (2 mg/kg) before the PBS challenge on day 13.

### LPS stimulation and salubrinal treatment in J744A.1 macrophages

J774A.1 macrophages were retrieved from the cell repository of Advanced Research Center, Central South University. Cells were cultured in a completed medium at 5% CO2 and 37 °C. There are four groups in vitro experiment: the control group (control), unstimulated cells; the LPS group (LPS), cells stimulated with LPS (10 ng/ml) for 24 h; the LPS + salubrinal groups, cells costimulated with salubrinal (10uM) and LPS (10 ng/ml) for 24 h; the salubrinal group, cells stimulated with salubrinal (10uM) for 24 h.

### Quantitative reverse transcription polymerase chain reaction (qRT-PCR)

Total RNA was extracted from cells with Trizol (Invitrogen). The cDNA was obtained using the reverse transcription kit (Vazyme). qRT-PCR was conducted according to the instructions of the manufacturer (Vazyme). The primers are displayed in Table 1.

**Table 1:** Primers for qRT-PCR.

Primers	Sequence (5′–3′)
Mouse DUSP2 forward	TGGTTCCAGGAGGCTATCA
Mouse DUSP2 reverse	CGAAGGAGGGAGAGCAAATAAG
Mouse CXCL10 forward	TGAGATCATTGCCACGATGAA
Mouse CXCL10 reverse	GAATTCTTGCTTCGGCAGTTAC
Mouse IL-1β forward	GCCCATCCTCTGTGACTCAT
Mouse IL-1β reverse	AGGCCACAGGTATTTTGTCG
Mouse TNF-α forward	CAGGCGGTGCCTATGTCTC
Mouse TNF-α reverse	CGATCACCCCGAAGTTCAGTAG
Mouse IL-17A forward	GAGAGCTTCATCTGTGTCTCTG
Mouse IL-17A reverse	GCATCTTCTCGACCCTGAAA
Mouse GAPDH forward	AAGGTCGGTGTGAACGGATT
Mouse GAPDH reverse	TGAGTGGAGTCATACTGGAACAT

### Flow cytometric analysis

The bronchoalveolar lavage (BAL) fluids were obtained by injecting 1 ml PBS into the murine trachea, repeating ten times. The cells of BAL fluid were stained with anti-CD45 (APC/Cy7; BioLegend), anti-CD11c (PE; BioLegend), anti-Siglec-F (APC; BioLegend), anti-MHCII (Percp/Cy5.5; BioLegend), anti-CD11b (BV605; BioLegend), anti-Ly6G (BV421; BioLegend), and anti-CD3 (AF488; BioLegend) from light for 30 min. The cell gating strategy in BAL fluids was conducted according to the methods previously reported^29^. The signals were obtained from a Cytek Dxp Athena flow cytometer. And the data were calculated using FlowJo software (version 10).

### Statistical analysis

All data were expressed as mean ± standard error (SE) and analyzed with SPSS 19.0 statistical software. The student’s t-test was performed to calculate the difference between the two groups. The one-way ANOVA followed by Tukey’s post hoc analysis was used to calculate the differences in more than two groups. The difference was significant when a *P* value was < 0.05.

## Results

### Differentially expressed genes

**Figure 1:**
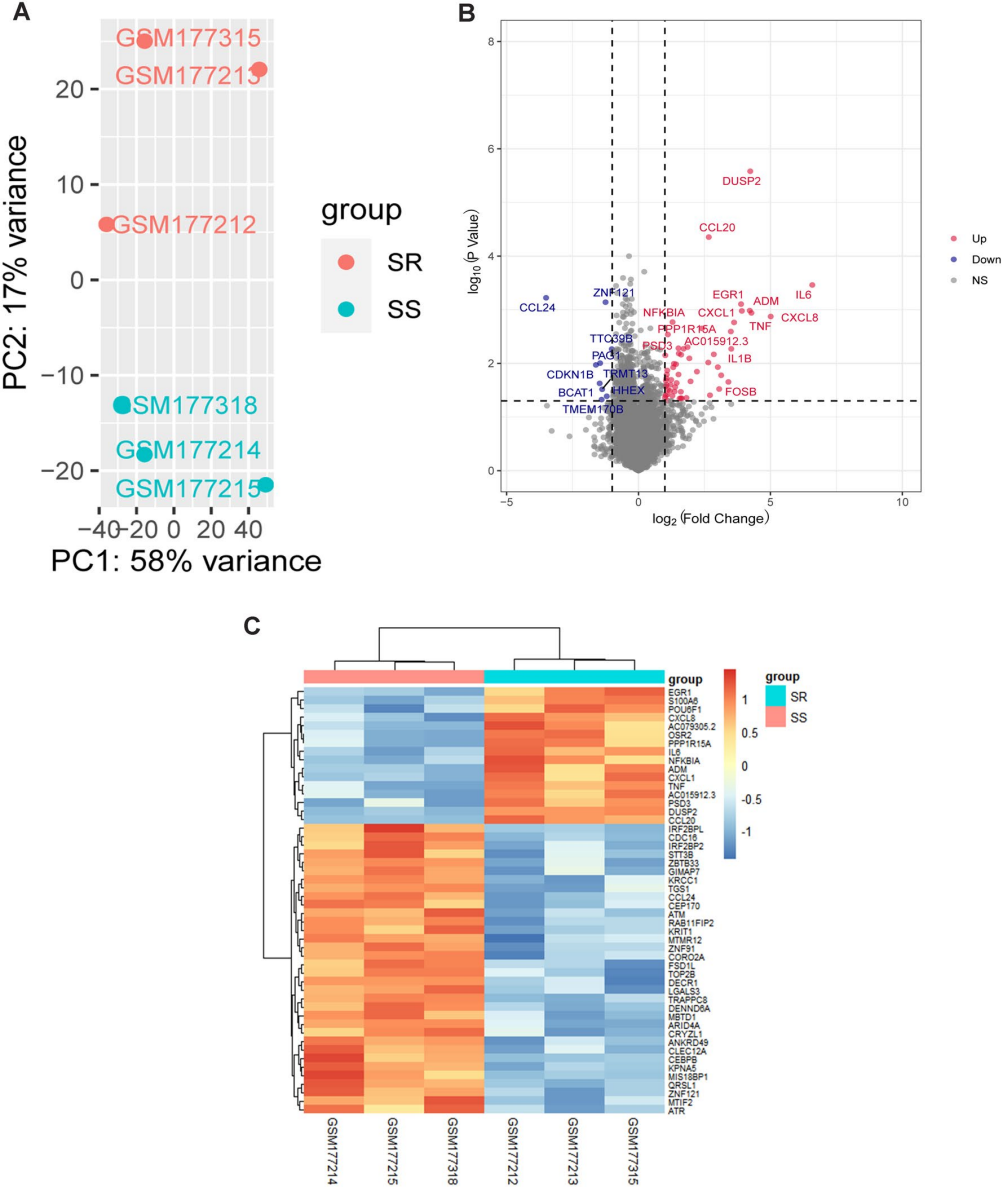
Profiles of expressed genes in patients with steroid-resistant asthma (SR) and steroid-sensitive (SS) asthma. (**A**) Principal-component analysis (PCA) among samples. (**B**) The volcano plot of gene expressions in SR patients and SS patients. The red dots and the blue dots indicate upregulated DEGs and downregulated DEGs, respectively. (**C**) A heatmap for the DEGs was constructed using pheatmap (version 1.0.12) packages in the R environment (version 3.6.2).

We analyzed the microarray dataset GSE7368 from the GEO database and selected DEGs between BALF cells from steroid-resistant asthma (SR) and steroid-sensitive (SS) asthma using R packages. Principal-component analysis displayed that the center of the SR group lay far apart from the center of the SS group in space, indicating the different expressions of genes between the two groups (Fig. 1A). A total of 57 upregulated and 9 downregulated genes were determined as DEG genes from SR patients and SS patients, as displayed in Table 2. The volcano plots showed significantly upregulated or downregulated DEGs in SR patients compared with SS patients (Fig. 1B). The red dots indicate the significantly upregulated genes, while the blue dots indicate the significantly downregulated genes. Hierarchical clustering heatmaps manifested the distinguishable DEGs expression pattern between SR patients and SS patients (Fig. 1C). These results above showed that the top 6 DEGs between SR patients and SS patients were IL6, CXCL8, TNF, DUSP2, ADM, and CXCL1.

**Table 2:** Differentially expressed genes.

Gene symbol	logFC	*P* value	Gene ID	Gene title	Location
Upregulated genes	SR-SS	SR-SS			
IL6	6.59	0.0003	ENSG00000136244	Interleukin 6	Chromosome 7
CXCL8	5.01	0.0013	ENSG00000169429	C-X-C motif chemokine ligand 8	Chromosome 4
TNF	4.28	0.0011	ENSG00000232810	Tumor necrosis factor	Chromosome 6
DUSP2	4.24	0.0000	ENSG00000158050	Dual specificity phosphatase 2	Chromosome 2
ADM	4.22	0.0010	ENSG00000148926	Adrenomedullin	Chromosom 11
CXCL1	3.92	0.0011	ENSG00000163739	C-X-C motif chemokine ligand 1	Chromosom 4
EGR1	3.89	0.0008	ENSG00000120738	Early growth response 1	Chromosom 5
AC079305.2	3.62	0.0017	Uncharacterized	Uncharacterized AC079305.2	Uncharacterized
IL1B	3.52	0.0054	ENSG00000125538	Interleukin 1 beta	Chromosom 2
AC015912.3	3.50	0.0025	Uncharacterized	Uncharacterized AC015912.3	Uncharacterized
FOSB	3.41	0.0222	ENSG00000125740	FosB proto-oncogene	Chromosom 19
SLPI	3.13	0.0167	ENSG00000124107	Secretory leukocyte peptidase inhibitor	Chromosom 20
PMAIP1	3.06	0.0300	ENSG00000141682	Phorbol-12-myristate-13-acetate-induced protein 1	Chromosom 18
NFKBIZ	3.01	0.0117	ENSG00000144802	NFKB inhibitor zeta	Chromosom 3
CXCL3	2.85	0.0068	ENSG00000163734	C-X-C motif chemokine ligand 3	Chromosom 4
PTGS2	2.71	0.0392	ENSG00000073756	Prostaglandin-endoperoxide synthase 2	Chromosom 1
CCL20	2.66	0.0000	ENSG00000115009	C–C motif chemokine ligand 20	Chromosom 2
TNFAIP3	2.64	0.0096	ENSG00000118503	TNF alpha induced protein 3	Chromosom 6
PPP1R15A	2.40	0.0022	ENSG00000087074	protein phosphatase 1 regulatory subunit 15A	Chromosom 19
ATP2B1-AS1	2.21	0.0143	ENSG00000271614	ATP2B1 antisense RNA 1	Chromosom 12
CD2	1.97	0.0216	ENSG00000116824	CD2 molecule	Chromosom 1
AC005083.1	1.93	0.0081	ENSG00000233834	AC005083.1	Chromosome 7
AL118516.1	1.86	0.0050	Uncharacterized	Uncharacterized AL118516.1	Uncharacterized
NINJ1	1.82	0.0440	ENSG00000131669	Ninjurin 1	Chromosom 9
IER2	1.71	0.0053	ENSG00000160888	Immediate early response 2	Chromosom 19
CAMP	1.64	0.0449	ENSG00000164047	Cathelicidin antimicrobial peptide	Chromosom 3
BCL10	1.61	0.0069	ENSG00000142867	BCL10 immune signaling adaptor	Chromosom 1
SNTN	1.60	0.0445	ENSG00000188817	Sentan, cilia apical structure protein	Chromosom 3
EID3	1.60	0.0338	ENSG00000255150	EP300 interacting inhibitor of differentiation 3	Chromosom 12
PHLDA1	1.56	0.0452	ENSG00000139289	Pleckstrin homology like domain family A member 1	Chromosom 5
GPX3	1.53	0.0065	ENSG00000211445	Glutathione peroxidase 3	Chromosom 5
ATF3	1.52	0.0161	ENSG00000162772	Activating transcription factor 3	Chromosom 1
THAP2	1.51	0.0052	ENSG00000173451	THAP domain containing 2	Chromosom 12
TCN2	1.44	0.0232	ENSG00000185339	Transcobalamin 2	Chromosom 22
OCIAD2	1.42	0.0104	ENSG00000145247	OCIA domain containing 2	Chromosom 4
NFKBIE	1.39	0.0277	ENSG00000146232	NFKB inhibitor epsilon	Chromosom 6
SOD2	1.36	0.0335	ENSG00000112096	Superoxide dismutase 2	Chromosom 6
AL359711.2	1.34	0.0101	Uncharacterized	Uncharacterized AL359711.2	Uncharacterized
PADI2	1.31	0.0118	ENSG00000117115	peptidyl arginine deiminase 2	Chromosom 1
NFKBIA	1.29	0.0017	ENSG00000100906	NFKB inhibitor alpha	Chromosom 14
ICAM1	1.28	0.0403	ENSG00000090339	Intercellular adhesion molecule 1	Chromosom 19
TACSTD2	1.24	0.0203	ENSG00000184292	Tumor associated calcium signal transducer 2	Chromosom 1
IL15RA	1.21	0.0250	ENSG00000134470	Interleukin 15 receptor subunit alpha	Chromosom 10
CERK	1.19	0.0324	ENSG00000100422	Ceramide kinase	Chromosom 22
PSD3	1.11	0.0029	ENSG00000156011	Pleckstrin and Sec7 domain containing 3	Chromosom 8
TMC5	1.11	0.0310	ENSG00000103534	Transmembrane channel like 5	Chromosom 16
ZFP36	1.10	0.0493	ENSG00000128016	ZFP36 ring finger protein	Chromosom 19
KLRB1	1.09	0.0136	ENSG00000111796	Killer cell lectin like receptor B1	Chromosom 12
CD80	1.08	0.0384	ENSG00000121594	CD80 molecule	Chromosom 3
TAGAP	1.07	0.0161	ENSG00000164691	T cell activation RhoGTPase activating protein	Chromosom 6
PNRC1	1.06	0.0251	ENSG00000146278	Proline rich nuclear receptor coactivator 1	Chromosom 6
ACVR2A	1.06	0.0190	ENSG00000121989	Activin A receptor type 2A	Chromosom 2
LRRC23	1.04	0.0209	ENSG00000010626	Leucine rich repeat containing 23	Chromosom 12
GBP2	1.03	0.0269	ENSG00000162645	Guanylate binding protein 2	Chromosom 1
RSPH1	1.03	0.0398	ENSG00000160188	Radial spoke head component	Chromosom 21
OSBPL3	1.02	0.0071	ENSG00000070882	Oxysterol binding protein like 3	Chromosom 7
SIGLEC10	1.00	0.0435	ENSG00000142512	Sialic acid binding Ig like lectin 10	Chromosom 19
Downregulated genes					
CCL24	− 3.50	0.0006	ENSG00000106178	C–C motif chemokine ligand 24	Chromosom 7
CDKN1B	− 1.62	0.0107	ENSG00000111276	Cyclin dependent kinase inhibitor 1B	Chromosom 12
BCAT1	− 1.47	0.0236	ENSG00000060982	Branched chain amino acid transaminase 1	Chromosom 12
PAG1	− 1.46	0.0099	ENSG00000076641	Phosphoprotein membrane anchor with glycosphingolipid microdomains 1	Chromosom 8
TMEM170B	− 1.40	0.0473	ENSG00000205269	Transmembrane protein 170B	Chromosom 6
TRMT13	− 1.38	0.0304	ENSG00000122435	tRNA methyltransferase 13 homolog	Chromosom 1
ZNF121	− 1.25	0.0007	ENSG00000197961	Zinc finger protein 121	Chromosom 19
HHEX	− 1.20	0.0410	ENSG00000152804	Hematopoietically expressed homeobox	Chromosom 10
TTC39B	− 1.01	0.0054	ENSG00000155158	Tetratricopeptide repeat domain 39B	Chromosom 9

### Tissue-specific expression of genes

We used BioGPS to identify 20 DEG genes as tissue-specific or organ system-specific expressed genes. Most of the tissue-expressed genes (65%, 13/20) were present in the hematologic/immune system. The respiratory and neurologic systems showed similar levels of tissue-expressed genes (10%, 2/20; 10%, 2/20), while the digestive, circulatory systems, and skin/skeletal muscle showed relatively low levels of tissue-expressed genes (5%, 1/20; 5%, 1/20; 5%, 1/20) (Table 3). Collectively, our results displayed that most tissue specific expressed DEG genes were distributed in the hematologic/immune system.

**Table 3:** Profiles of tissue-specific expressions of genes.

System	Genes
Hematologic/immune	DUSP2, EGR1, FOSB, PMAIP1, NFKBIZ, TNFAIP3, PPP1R15A,
	IER2, CAMP, NFKBIE, KLRB1, TAGAP, GBP2
Lung	ADM, SLPI
Neurologic	PADI2, PSD3
Digestive	TMC5
Circulatory	TCN2
Skin/skeletal muscle	PTGS2

### Functional and pathway enrichment analyses of DEGs

**Figure 2:**
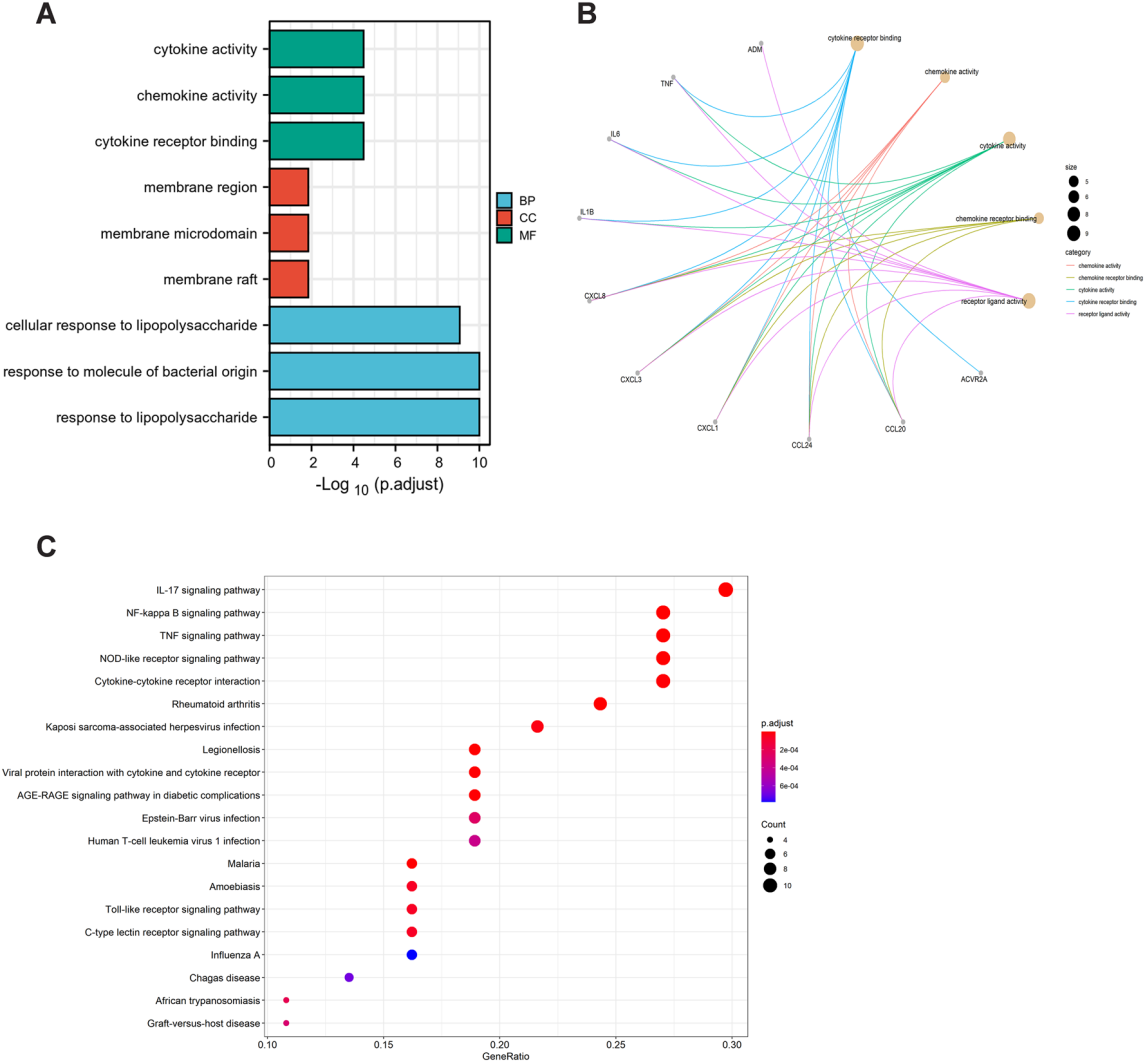
The results of GO and KEGG enrichment analysis of DEGs between SR patients and SS patients. (**A**) Bar plot of the functional GO terms, including biological process (BP), cellular component (CC), and molecular function (MF); (**B**) Circle graph showing the relationship between GO enrichment and DEGs. (**C**) Bar plot of KEGG enriched terms colored by a P value. The terms with more genes indicate a more significant P value.

To explore the function of DEGs between SR patients and SS patients, we performed GO and KEGG enrichment analyses. The biological processes (BPs) of DEGs were mainly enriched in cytokine activity, chemokine activity, and cytokine receptor binding. The cellular components (CCs) of DEGs were mainly involved in the membrane region, membrane microdomain, and membrane draft. The molecular functions (MFs) of DEGs were mainly enriched in the cellular response to lipopolysaccharide, response to the molecule of bacterial region, and response to lipopolysaccharide (Fig. 2A). The DEGs enriched in GO function enrichment were visualized using the ClusterProfiler package. The yellow dots indicated the GO categories, the color of the line indicated the association between dots and the category, and the size of a dot showed the gene numbers (Fig. 2B). As for the KEGG pathway enrichment analysis, DEGs were enriched in the IL-17 signaling pathway, NF-kappa B signaling pathway, TNF signaling pathway, NOD-like receptor signaling pathway, and cytokine-cytokine receptor interaction (Fig. 2C). Our results showed that the functional and pathway enrichment of DEGs was mainly related to cytokine responses.

### Gene set enrichment analysis

**Figure 3:**
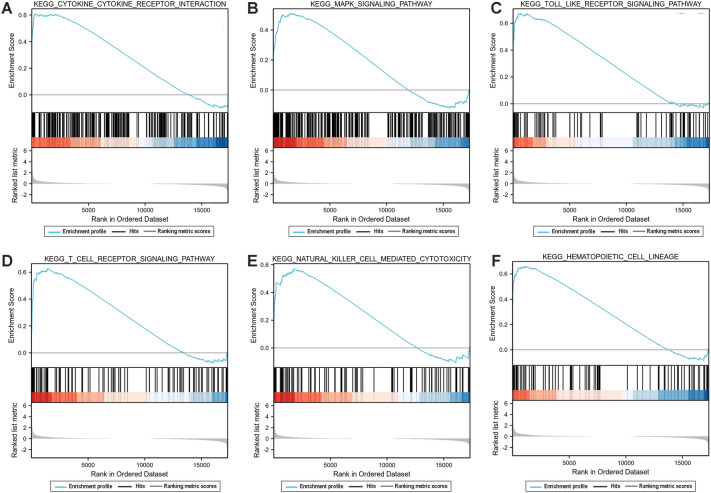
Gene set enrichment analysis (GSEA) of the enriched genes in the SR and SS group. (**A**) cytokine cytokine receptor interaction; (**B**) MAPK signal pathway; (**C**) Toll-like receptor signaling pathway; (**D**) T cell receptor signaling pathway; (**E**) Natural killer cell mediated cytotoxicity; (**F**) Hematopoietic cell lineage pathway.

The GSEA analysis was performed to explore the enriched gene-related pathways. The pathways related to cytokine cytokine receptor interaction, MAPK signal pathway, Toll-like receptor signaling pathway, T cell receptor signaling pathway, natural killer cell mediated cytotoxicity, and hematopoietic cell lineage pathway were mainly enriched in the SR group (Fig. 3A–F). Our results suggested that the enriched gene-related pathways were mainly associated with cytokine or immune responses.

### PPI networks of DEGs between SR patients and SS patients

**Figure 4:**
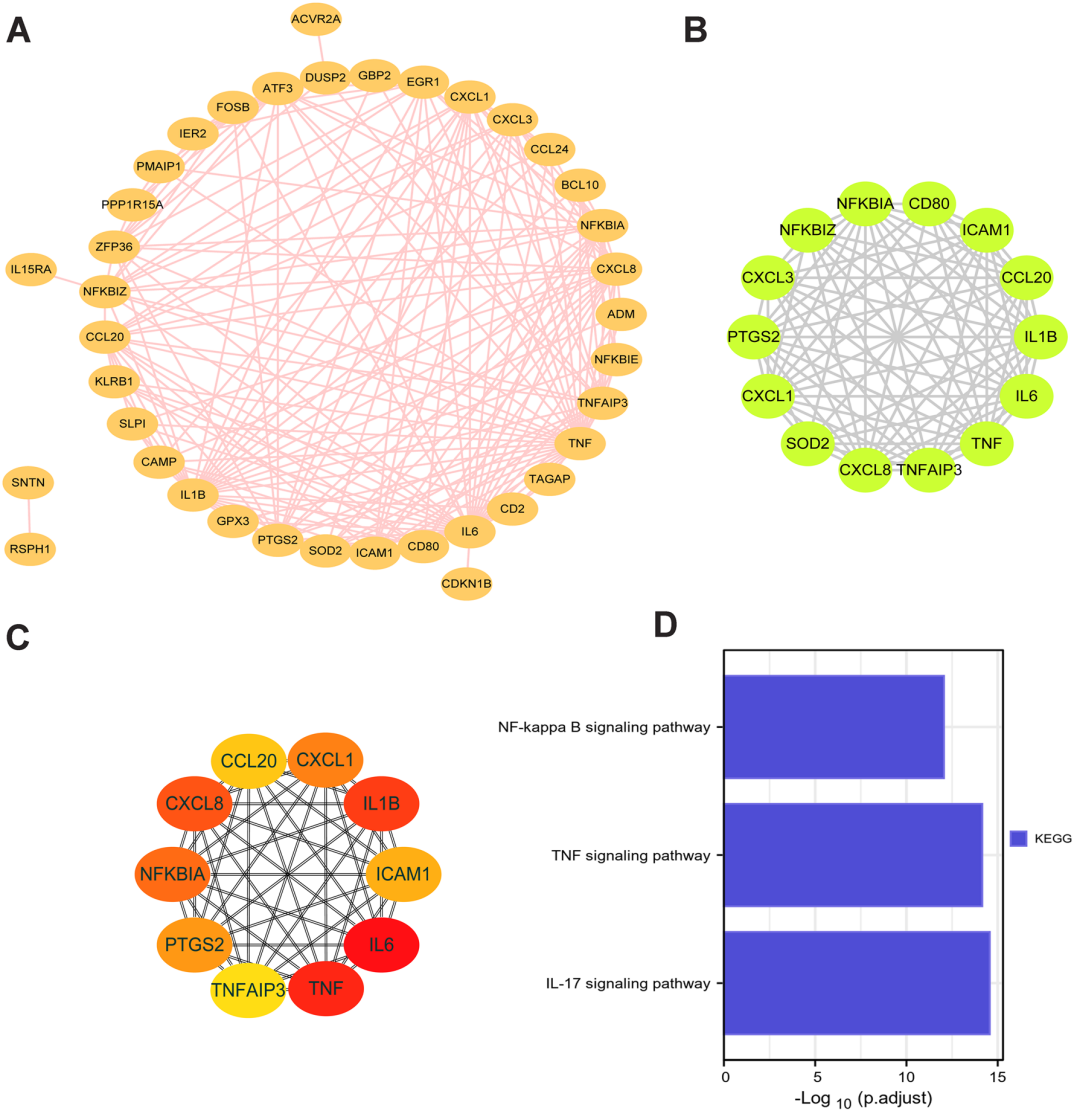
PPI Networks of DEGs. (**A**) The PPI network of DEGs was constructed using the STRING online database. (**B**) The key module with the highest score was identified by the MCODE in Cytoscape. (**C**) The top 10 hub gene cluster constructed by the Cytohubba in Cytoscape. (**D**) Functional enrichment analysis for the top 10 hub genes.

The protein association networks of DEGs between SR patients and SS patients were constructed by STRING. A PPI network with 60 nodes and 191 edges had an interaction score > 0.4 visualized by Cytoscape software (Fig. 4A). The nodes indicate genes, and the edges indicate the associations between genes. We used the MCODE plugin in Cytoscape to identify the key PPI network module with the highest score, consisting of 14 genes (Fig. 4B). Furthermore, the top 10 hub genes were identified by the Cytohubba in Cytoscape (Fig. 4C). The functional enrichment analysis showed that these top 10 hub genes were primarily related to the IL-17 signaling pathway, TNF signaling pathway, and NF-kappa B signaling pathways (Fig. 4D). These results revealed that DEGs between SR patients and SS patients were more likely to be related to inflammatory cytokine response.

### Validation of DUSP2 in an asthma mouse model

**Figure 5:**
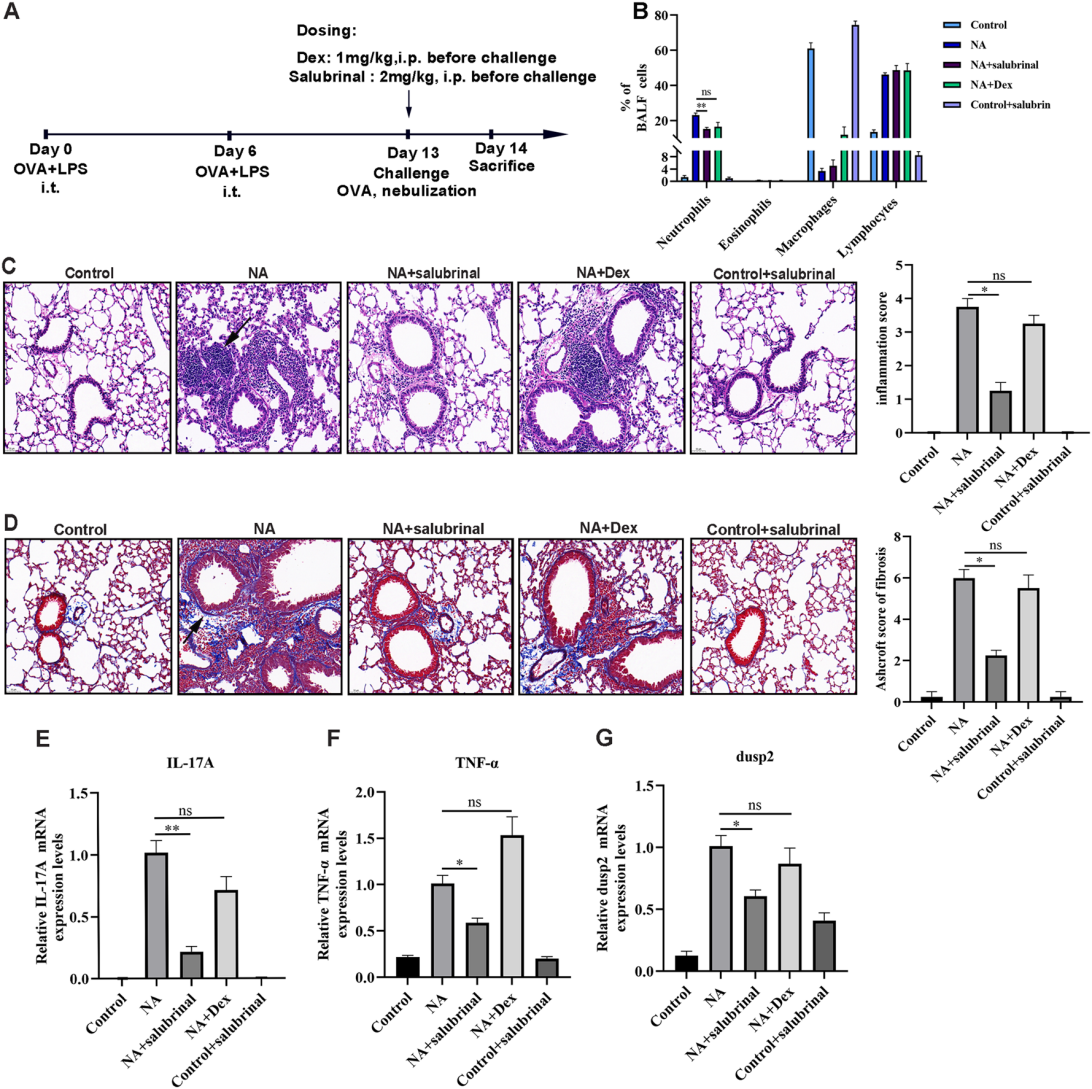
Validation of DUSP2 in an asthma mouse model. (**A**) Flow chart for a neutrophilic asthma mouse model establishment. (**B**) Inflammatory cells in BAL fluids. (**C**) Representative histological analysis of lung sections with H&E staining and a bar graph displaying the inflammation scores. Scale bar = 50 μm. (**D**) Representative histological analysis of lung sections with Masson staining and a bar graph displaying the Ashcroft scores. Scale bar = 50 μm. (**E**) Real-time PCR of IL-17A mRNA levels normalized to GAPDH. (**F**) Real-time PCR of TNF-α mRNA levels normalized to GAPDH. (**G**) Real-time PCR of DUSP2 mRNA levels normalized to GAPDH. n = 4, data were expressed as means ± SEM. *P < 0.05, **P < 0.01, ***P < 0.001. NA neutrophilic asthma.

Our analysis indicated that DUSP2 was potentially associated with steroid-resistant asthma. To our knowledge, there are few studies relating to the roles of DUSP2 in steroid-resistant asthma. Therefore, we hypothesized that inhibition of DUSP2 showed a protective effect on the airway inflammation of steroid-resistant asthma. We generated a steroid-resistant NA mouse model and administrated salubrinal according to the experimental procedure shown in Fig. 5A. It was demonstrated that more neutrophil infiltrations in BAL fluids were observed in the steroid-resistant murine asthma model^30^. Our study suggested that neutrophil numbers were not decreased by Dex treatment in the NA group (LPS/OVA + Dex) but were reduced after salubrinal administration in the NA group (LPS/OVA + salubrinal) (Fig. 5B). No significant changes in eosinophil, macrophage, and lymphocyte numbers of BAL fluids were observed in the NA group with or without salubrinal treatment (Fig. 5B). Besides, our study revealed that peribronchial inflammation infiltration and subepithelial collagen deposition were more severe in the NA group compared to the control group, and Dex failed to reverse lung morphological changes based on the inflammation score and Ashcroft score of fibrosis (Fig. 5C,D). As mentioned in the bioinformatic analysis above, DEGs were mainly related to IL-17 and TNF pathways. As a result, our study displayed that the NA group had the highest mRNA levels of IL-17A, TNF-α, and DUSP2 (Fig. 5E–G). The mRNA levels of IL-17A, TNF-α, and DUSP2 were not reduced by Dex administration in the NA group but were significantly reduced after salubrinal treatment in the NA group (Fig. 5E–G). These results indicated that the DUSP2 inhibitor ameliorated neutrophilic airway inflammation and cytokine responses (IL-17A and TNF-α)in an asthma mouse model with steroid-resistant.

### Inhibition of inflammatory cytokines by salubrinal in LPS-stimulated J744A.1 macrophages

**Figure 6:**
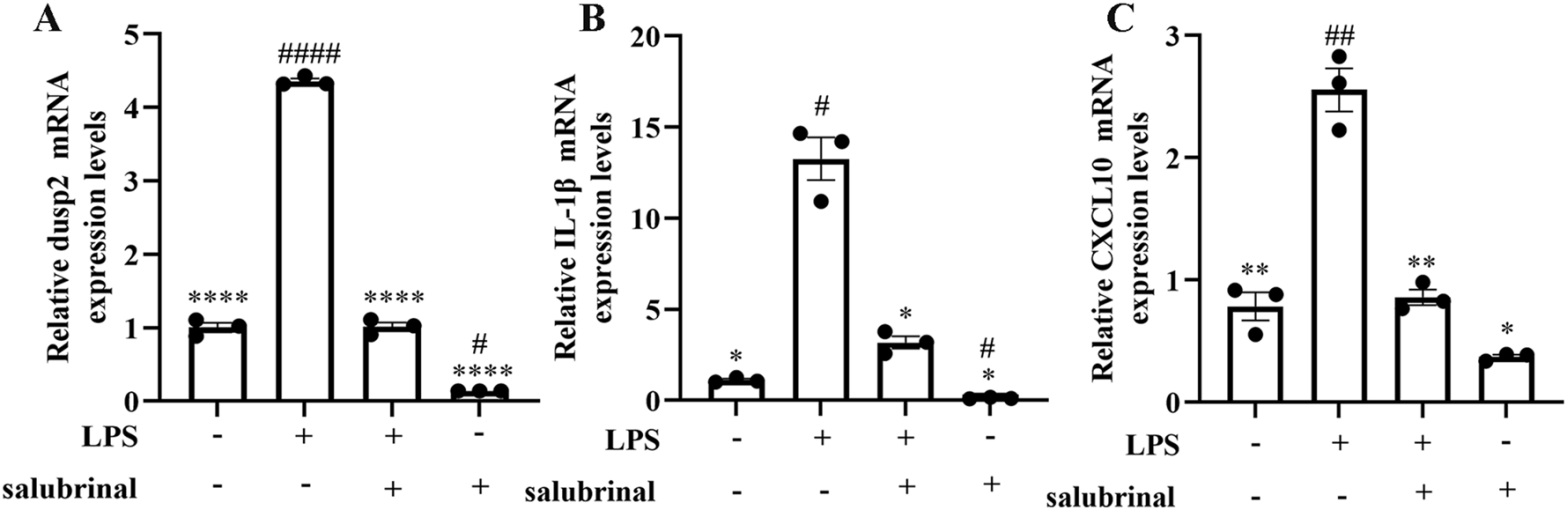
Inhibition of inflammatory cytokines by salubrinal in LPS-stimulated J774A.1 macrophages. (**A–C**) Effects of salubrinal on DUSP2, IL1β, and CXCL10 mRNAs in J774A.1 macrophage, respectively. n = 3. Data were expressed as means ± SEM. *P < 0.05, **P < 0.01, ***P < 0.001, vs LPS group. #*P* < 0.05, ##*P* < 0.01, ###*P* < 0.001, vs control group.

Incubation of J774A.1 macrophages with salubrinal significantly inhibited DUSP2 mRNA (Fig. 6A). Furthermore, the elevation of inflammatory cytokines (CXCL10, IL-1β) in LPS-stimulated J774A.1 macrophages was suppressed by salubrinal (Fig. 6B,C). Taken together, our results revealed that the correlation between DUSP2 and inflammatory cytokines (CXCL10, IL-1β) may be involved in the inflammatory response of LPS-stimulated J744A.1 macrophages.

## Discussion

Increased morbidity is observed in steroid-resistant asthma patients, and steroid-resistant asthma is more likely to be related to neutrophilic inflammation^5,31,32^. In this respect, it is urgent to explore alternative treatment options for steroid-resistant asthma. Our computational data-mining analysis explored several genes associated with steroid-resistant asthma, aiming to provide new insight into the pathogenesis of steroid-resistant asthma. Different from the original source of the data set, our study pays more attention to the evaluation and correction of data. We use the limma package to search for differential genes, thereby improving the power of the test.

In the current study, we conducted a discovery-driven analysis to identify DEGs in cells of BAL fluids from SR asthma patients and SS asthma patients. 66 DEGs were identified, consisting of 57 upregulated genes and 9 downregulated genes, which were consistent with activated macrophage-related genes. The top six upregulated DEG genes (IL6, CXCL8, TNF, DUSP2, ADM, and CXCL1) in SR asthma patients compared with SS asthma patients were identified. Recent studies have indicated that reduced expression of IL6 suppressed the development of steroid asthma^33,34^. CXCL8, also named IL8, is a CXC chemokine that could recruit neutrophils in steroid-resistant airway inflammation^35,36^. Dex could not reduce the levels of IL6, and IL8 in the macrophages of severe asthma patients^37^. It was found that patients with steroid-resistant showed a higher level of TNF-α than patients with steroid-sensitive in C. pneumoniae seropositive asthmatics^38^. Dex could not suppress the levels of TNF-α in steroid-resistant asthma mouse models^32^. LIGHT and LTαβ, which belong to the TNF family, were reported to induce a steroid-resistant inflammatory response in airway epithelial cells^39^. DUSP2, also named phosphatase of activated cells 1 (PAC-1), was reported to function as dephosphorylating threonine and tyrosine residues of targeting substrates^40,41^. DUSP2, predominantly expressed in hematopoietic cells, was demonstrated to upregulate the production of proinflammatory cytokines in immune cells^42,43^. However, few studies have investigated the role of DUSP2 in steroid-resistant asthma. It is necessary to further delineate the pathogenesis role of DUSP2 in steroid-resistant asthma. One study has revealed that plasma ADM was increased in the acute attack period of asthma compared to the remission period of asthma^44^, suggesting ADM may be involved in the process of asthma. CXCL1 was observed to be a kind of neutrophil-related chemokine^45^, which was elevated in neutrophilic asthma patients^46^. Immune cells were reported to play important roles in the pathogenic development of asthma, and asthma is characterized by granulocytic inflammation infiltration in the airways^44,47^. In our study, the tissue-specific expression analysis indicated that the hematologic/immune system was the most highly specific system of the DEGs, suggesting the common occurrence of immune response in steroid-resistant asthma.

Enrichment analyses were constructed to explore the roles of DEGs. Results from the GO analysis revealed that the DEGs were mainly enriched in cytokine activity, chemokine activity, cytokine receptor binding, membrane region, membrane microdomain, membrane draft, cellular response to lipopolysaccharide, response to molecule of the bacterial region, and response to lipopolysaccharide. Cytokine-cytokine receptor interaction was reported to be more enriched in neutrophilic asthma than in eosinophil asthma^48^. Lipopolysaccharide (LPS), a component of gram-negative bacteria, was reported to chemoattract neutrophils^49^. These biological pathways are mainly associated with an inflammatory response. KEGG analysis demonstrated that the DEGs were mainly enriched in the IL-17 signaling pathway, NF-kappa B signaling pathway, TNF signaling pathway, NOD-like receptor signaling pathway, and cytokine-cytokine receptor interaction. After analyzing the PPI network of DEGs, we found that the top 10 hub genes identified by the Cytohubba in Cytoscape were primarily enriched in the IL-17 signaling pathway, TNF signaling pathway, and NF-kappa B signaling pathways, which were consistent with the KEGG enrichment analysis. IL-17 signaling pathway has been suggested to mediate the pathogenesis of steroid-resistant asthma, which has the function of neutrophil chemotaxis^50,51^. Nuclear factor-κB (NF-κB) was reported to lead to epithelial cell inflammation of asthma and may be a potential marker for asthma severity^52^. Tumor necrosis factor-alpha (TNF-α) was reported to be associated with steroid-resistant asthma and asthma exacerbation frequency^53,54^. These functional enrichment analyses may exhibit the enrichment of DEGs in a neutrophil-related inflammatory response in steroid-resistant asthma. Different from the previous study^55^, our study has added the enrichment analyses of DEG genes, which suggested that neutrophil-related inflammatory responses were implicated in steroid-resistant asthma.

The GSEA data revealed that cytokine cytokine receptor interaction, MAPK signal pathway, Toll-like receptor signaling pathway, T cell receptor signaling pathway, natural killer cell mediated cytotoxicity, and hematopoietic cell lineage pathway were mainly enriched in SR patients. The p38 MAPK pathway activation was reported to be involved in steroid resistance of asthma^56^. Dex failed to inhibit NTHi-induced steroid-resistant allergic airway inflammation, by activating the p38 MAPK pathway^57^. Immune cells were involved in the pathogenic process of asthma^44^. More than half of the pulmonary CD4^+^CD3^+^ cells in moderate-to-severe asthma patients were natural killer T cells, with elevated invariant T-cell receptor expression^58^. Toll-like receptors (TLRs) were reported to be involved in macrophage-related steroid-resistant AHR^59,60^. The modulation of the T-cell receptor (TCR) in CD4^+^T cells recruited inflammatory cells and elevated cytokine production in the airway of asthma^61^. Our findings support the association between immune cell activation, inflammatory response, and steroid-resistant asthma.

The DUSP2 gene was reported to be involved in the immune activation process, which could enhance inflammatory responses^43^. However, few studies have reported the role of DUSP2 in steroid-resistant asthma. Since our study suggested that DUSP2 was upregulated in the BAL cells of SR asthma patients as compared with SS asthma patients, we further investigate the role of DUSP2 on steroid-resistant asthma. Previous studies have suggested that salubrinal was a candidate drug for inhibiting the expression of DUSP2^62,63^. Salubrinal is a 480-Da agent that inhibits a serine phosphatase, protein phosphatase 1 (PP1) and elevates the phosphorylation of eukaryotic translation initiation factor 2 alpha (eIF2α)^64^. It was suggested that LPS significantly increased DUSP2 expression but caused a decrease in the p-eIF2a/eIF2a ratio, which was reversed by salubrinal in the LPS-induced intraneural hemi-Parkinson disease (PD) model^62^. Through eIF2α, salubrinal could mediate transcriptional and translational regulation^63,65^. Therefore, we can assume that salubrinal may inhibit the transcriptional and translational process of DUSP2 by acting on p-eIF2a/eIF2a ratio. It was found that the administration of salubrinal in mouse models of arthritis significantly reduced inflammatory responses^63^. Interestingly, our findings revealed that administration of a candidate synthetic DUSP2 inhibitor (salubrinal) before the challenge reduced the neutrophilic airway inflammation and cytokine responses (IL-17A, TNF-α) of the steroid-resistant asthma mouse model, suggesting a protective effect of salubrinal in steroid-resistant asthma. Macrophages are considered to be the main immune cells in asthma^66^. Results from a previous study indicated that salubrinal reduced the DUSP2 expressions in both RAW264.7 macrophages and Jurkat cells^63^. In line with the previous studies, our study supports that salubrinal is an inhibitor of DUSP2. It was founded that salubrinal inhibited inflammatory cytokines (IL-1β, IL-6, TNF-α), and DUSP2 expressions stimulated by LPS^62,63^. CXCL10 is a kind of inflammatory cytokine, which enhanced type 1 inflammation resulting in severe pathology^67^. IL-1β was reported to induce neutrophilic inflammation^68^. Of note, CXCL10 and IL-1β were reported to be elevated in steroid-resistant asthma^30,69,70^. Consistent with the previous studies, our study revealed that salubrinal inhibited LPS-driven DUSP2 and IL-1β expressions in J774A.1 macrophage. Besides, we first demonstrated that salubrinal reduced LPS-driven CXCL10 expression in J774A.1 macrophage, indicating the interaction between DUSP2 and CXCL10 may be the potential mechanism for steroid-resistant asthma.

## Limitations

That said, there are some limitations of the present study. First, the sample size of asthma patients in the public datasets was small. More BAL cell expression profiles of asthmatics will be needed in future analysis. Second, more potential in vitro mechanisms should be determined in further study.

## Conclusion

In conclusion, several DEGs and enriched pathways in the SR patients compared to the SS patients were identified. Our study not only provides an insight into the role of DUSP2 and its inhibitor (salubrinal) in steroid-resistant neutrophilic airway inflammation but also lay a foundation for the development of alternative therapy options in steroid-resistant asthma.

## Data Availability

The datasets generated for this study can be found in the GEO dataset: https://www.ncbi.nlm.nih.gov/geo/query/acc.cgi?acc=GSE7368. The permission is granted to (Springer Nature Limited) to publish the images under a CC BY open access license and in all formats i.e. print and digital.

## Abbreviations

GEO: Gene Expression Omnibus
DEGs: Differentially expressed genes
SR: Steroid-resistant
SS: Steroid-sensitive
PPI: Protein–protein interaction
LPS: Lipopolysaccharide
OVA: Ovalbumin
qRT-PCR: Quantitative reverse transcription-polymerase chain reaction
GCs: Glucocorticoids
BALF: Bronchoalveolar lavage fluid
BP: Biological process
CC: Cellular component
MF: Molecular function
FDR: False discovery rate
MCODE: Molecular Complex Detection
NA: Neutrophilic asthma
H&E: Hematoxylin and eosin
Dex: Dexamethasone
SD: Standard deviation
NF-κB: Nuclear factor-κB
TNF-α: Tumor necrosis factor-alpha
NLRP3: Nod-like receptor family pyrin domain containing 3
TLRs: Toll-like receptors
